# Nonlinear network model analysis of vibrational energy transfer and localisation in the Fenna-Matthews-Olson complex

**DOI:** 10.1038/srep36703

**Published:** 2016-11-09

**Authors:** Sarah E. Morgan, Daniel J. Cole, Alex W. Chin

**Affiliations:** 1 Theory of Condensed Matter Group, Physics Department, University of Cambridge, CB3 0HE, United Kingdom; 2 School of Chemistry, Newcastle University, Newcastle upon Tyne, NE1 7RU, United Kingdom

## Abstract

Collective protein modes are expected to be important for facilitating energy transfer in the Fenna-Matthews-Olson (FMO) complex of photosynthetic green sulphur bacteria, however to date little work has focussed on the microscopic details of these vibrations. The nonlinear network model (NNM) provides a computationally inexpensive approach to studying vibrational modes at the microscopic level in large protein structures, whilst incorporating anharmonicity in the inter-residue interactions which can influence protein dynamics. We apply the NNM to the entire trimeric FMO complex and find evidence for the existence of nonlinear discrete breather modes. These modes tend to transfer energy to the highly connected core pigments, potentially opening up alternative excitation energy transfer routes through their influence on pigment properties. Incorporating localised modes based on these discrete breathers in the optical spectra calculations for FMO using ab initio site energies and excitonic couplings can substantially improve their agreement with experimental results.

The Fenna-Matthews-Olson complex (FMO) is an archetypal light-harvesting system, found in green sulphur bacteria. FMO acts as a funnel, transferring energy from the light harvesting antennae to the reaction centre. It has a trimeric structure in which each monomer contains 8 bacteriochlorophyll-a pigments (BChla)^1^. FMO has attracted considerable attention recently due to the long-lasting oscillations which have been observed in 2D spectroscopy of the complex even at room temperature^2^,^3^,^4^. These oscillations were originally assigned to picosecond-long electronic coherences^4^, which created tremendous interest in the potential role of quantum effects in biological light harvesting systems^5^, although later work has ascribed the oscillations to ground state vibrations^6^. Meanwhile, there is growing evidence that vibrational modes play an important role in excitonic dynamics, and these vibronic effects may arise either from intra-pigment or protein motions^7^,^8^,^9^,^10^,^11^,^12^. Nonetheless, to date the protein part of the environment has not been well characterised and the spatial localisation of the protein vibrations remains unclear. This gap in the literature is primarily due to the computational cost and complexity of studying these modes in all atom models.

Vibrational dynamics in proteins have attracted attention in a range of contexts^13^,^14^,^15^,^16^,^17^,^18^ and experimental methods are emerging which can probe these dynamics^19^,^20^. Protein vibrations tend to be much less rigid than intramolecular pigment modes and can remain out of equilibrium over long time periods^21^, hence non-linear effects are often important^22^,^23^. In addition, protein modes can have long lifetimes, as observed in IR experiments^19^,^24^, for example oscillations which last for over 500 ps in bacteriorhodopsin^24^. As well as experimental results, vibrational modes and vibrational energy transfer have also been studied in all atom computational models^13^,^14^,^18^. Renger *et al.* considered the normal modes of FMO using an atomistic model^25^, although their work focussed on deriving the spectral function and they did not discuss the spatial properties of individual modes or anharmonic effects. Moreover, FMO’s large size meant that Renger *et al.* were only able to study a single monomer. Whilst there is only weak excitonic coupling between FMO’s monomers, we will show that the trimeric structure can be important for mechanical effects.

Cheaper computational methods have been employed to overcome this size limitation, both in FMO and in other proteins, including coarse-grained elastic network models and normal mode analysis (NMA). We note that typical parameters for these models put the highest frequency normal modes in the region of 100 cm^−1^, which is at the boundary of inter-exciton gaps in FMO and overlaps with intramolecular modes of the pigments that could be coupled to excitons. The high frequency region of protein phonons could therefore be an important mediator for energy transfer between FMO’s local optoelectronics and its wider structure. In this work, our aim is to study the properties of FMO’s vibrational modes in this region, which we note is intermediate between low frequency conformational protein fluctuations (with frequencies of a few cm^−1^ or less) and high frequency bond vibrations (which can have frequencies of thousands of cm^−1^ and are not generally included in coarse-grained models). Since the spatial localisation of vibrational modes tends to increase with mode frequency^13^, the localisation of modes with frequencies in the region of 100 cm^−1^ is also intermediate between these two extremes, and can be on the scale of a few residues. Here we are interested in the spatial localisation of these modes in FMO.

In general, work using cheaper computational methods tends not to include anharmonicity and to focus on the lowest frequency modes (≈10 cm^−1^)^16^,^15^. However, in their work on the normal harmonic modes of the FMO monomer, Renger *et al.* found that phenomenological corrections were needed to account for the strong anharmonicity of the low frequency normal modes and obtain good agreement with the low-frequency spectral density known from experiments^25^. The nonlinear network model (NNM) proposed by Juanico *et al.*^26^ offers a promising approach to explicitly model nonlinear protein dynamics in a computationally inexpensive way. Interestingly, previous studies applying this model to a number of proteins have found evidence for the spontaneous localisation of energy and formation of discrete breather modes (DBs)^27^,^28^,^29^. These localised vibrational modes are able to harvest energy from their surroundings and to transfer energy between different protein residues on picosecond timescales. Whilst most work on DBs to date is theoretical, correlated motions in ubiquitin revealed by experimental NMR measurements match the calculated displacement patterns of DBs^30^, suggesting that simple DB models are able to capture some experimental features. Nonlinearity can also play an important role in redistributing energy amongst modes, which cannot be captured by any harmonic picture. If supported by light harvesting pigment protein complexes, DBs could therefore be functionally important for targeted vibrational energy transfer and storage^31^.

Crucially, previous work suggests that DBs form in interface regions of a protein that are rigid and away from surfaces, which are more flexible and exposed to damping. FMO is exceptionally rigid^32^ and - being a trimer - contains a highly shielded interface at its core, which contains several of the most important pigments (the 7 pigments within each protein envelope are shown in Fig. 1a). FMO is therefore an attractive candidate to explore the concepts and phenomenology of DBs in proteins.

In the following, we begin by studying the high frequency normal modes (NMs) of FMO, several of which are delocalised across the trimer and have strong components on the pigments in the core of the FMO structure. Note that here we use the term ‘high frequency modes’ to refer to high frequency modes in our model, which are found near the top of the normal mode spectrum. These modes have frequencies of around 100–200 cm^−1^, since our coarse-grained model does not contain bond vibrations etc. We then compute the dynamics which follow excitation of the highest frequency NM and find evidence for the existence of DBs in the structure. The DB which forms following excitation of the highest frequency NM is localised close to pigments 3, 4 and 7 and is therefore most likely to modulate the site energies of those pigments.

An additional motivation for the current work is a growing recognition that parameters of the pigment-protein Hamiltonian, namely pigment site energies and excitonic coupling strengths, may be extracted directly from the X-ray crystal structure. Work in this area tends to focus on the FMO complex and ranges in complexity from fully quantum mechanical (QM) simulations^33^,^34^ to models in which the protein environment is treated using classical molecular mechanics point charges (QM/MM)^35^,^36^. If successful, these methods could potentially guide studies of much larger complexes for which fitting of the site energies to experimental data becomes infeasible.

Cole *et al.*^33^ used large-scale density functional theory calculations to extract parameters of the FMO complex directly from the crystal structure. They then used their results to simulate the optical spectra (in particular the linear absorption (LA), linear dichroism (LD) and circular dichroism (CD) spectra). Good agreement was obtained with the experimental spectra at low frequencies but there were substantial discrepancies at higher frequencies. However, it was unclear whether these discrepancies were due to a limitation in the quantum mechanical model or in the spectral density, which was modelled using an Ohmic spectral density function which was assumed to be identical on all sites. Therefore in the last part of this work, we study the effect which a localised DB mode might have on the optical spectra. To do this, we use the pigment energies and couplings obtained by Cole *et al.*^33^ from first principles using large scale quantum-mechanical calculations. We then approximate the DB with a mode of frequency *ω* = 100 or 180 cm^−1^, which couples to pigments 3, 4 and 7. We show that the mode can substantially improve the agreement between the calculated and experimental LA and LD spectra.

## Methods

We model FMO using the NNM introduced by Juanico *et al.*^26^. In this model each amino acid is represented by a point-like node of mass 110 atomic mass units, which is placed at the position of the corresponding C_*α*_ atom. Calculations were based on the holo (8 BChla per monomer) form of the trimeric 1.3 Å X-ray crystal structure of *Prosthecochloris aestuarii* (PDB: 3EOJ)^37^, although as mentioned above we only include 7 pigments in our network model. Based on their relative masses, the chlorin ring and phytol tail of the BChla pigment molecules were modelled by 5 nodes and 3 nodes, respectively. The nodes were placed at the positions of the MG, C2A, C2B, C2C, C2D, C2, C10 and C18 atoms (crystal structure atom labelling). Together the residues and pigment atoms make up the 1242 nodes of our model. We neglect the effect of the environment because FMO has an extremely rigid clam shell structure^32^ and, as discussed in the results and discussion section, the nonlinear modes we observe are localised around the pigments in the centre of the FMO structure (in particular pigments 3, 4 and 7). Therefore we expect them to be effectively shielded from the environment by the clam shell. More generally, we would expect the environment to act mainly as a source of boundary friction that effectively dissipates any modes with displacements near the surface^26^,^29^,^38^.

The NNM potential energy has both a linear and a nonlinear term and reads^26^,^30^:
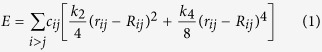
where 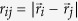 is the instantaneous distance between nodes i and j and 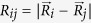 is the distance between nodes i and j at equilibrium (t = 0). *c*_*ij*_ = 1 if *R*_*ij*_ is less than a cut-off distance, *R*_*c*_, otherwise *c*_*ij*_ = 0. We set *R*_*c*_ = 10 Å, *k*_2_ = 10 kcal/mol/Å^2^ and *k*_4_ = 10 kcal/mol/Å^4^ unless otherwise stated. These values have been used in previous studies of the NNM and have been shown to reproduce several important features of NMR protein experiments^26^,^30^. We note that these previous works have also motivated the NNM as arising from an effective Taylor expansion of a “potential of mean force” between carbon alpha atoms in the network, which could open new routes to extracting protein-specific *k*_2_ and *k*_4_ in the future^26^,^30^.

We obtain the normal modes of the system by calculating the eigenvectors and eigenvalues of the Hessian matrix. We then perform molecular dynamics simulations using a Verlet algorithm with a 1 fs time step. The network is excited with total energy *E*_0_ in the direction of one of the eigenvectors (as specified in the text). We record the total energy of every residue at each time step, given by a sum of the kinetic and potential energies.

In order to calculate the optical spectra, we use the pigment energies and couplings obtained by Cole *et al.* from large-scale quantum mechanical calculations^33^. We also couple pigments 3, 4 and 7 to a single vibrational mode in order to represent the observed DBs, which is incorporated on an equal footing with the electronic energy levels. The total Hamiltonian reads:
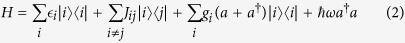
where 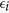 is the site energy of pigment i, *J*_*ij*_ is the coupling between optical excitations on pigments i and j, *ω* is the mode frequency and *g*_*i*_ is the coupling of pigment i to the vibrational mode. *g*_*i*_ depends on the Huang Rhys factor, S, according to 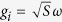. To calculate the spectra we use the Master equation approach taken by Renger and Marcus^39^ and Cole *et al.*^33^, which makes the Markov approximation and hence each excitonic transition has a Lorentzian lineshape. We use the same parameters as Cole *et al.* for the pure dephasing rate and the spectral density function (which provides a smooth background bath)^33^. To include the effect of static disorder, the final spectra are obtained by averaging over 1000 realisations of the site energies, each drawn from a Gaussian distribution with a full-width half maximum of 100 cm^−1^. We include 15 harmonic oscillator levels for the mode, which gives converged spectra.

## Results and Discussion

**Figure 1:**
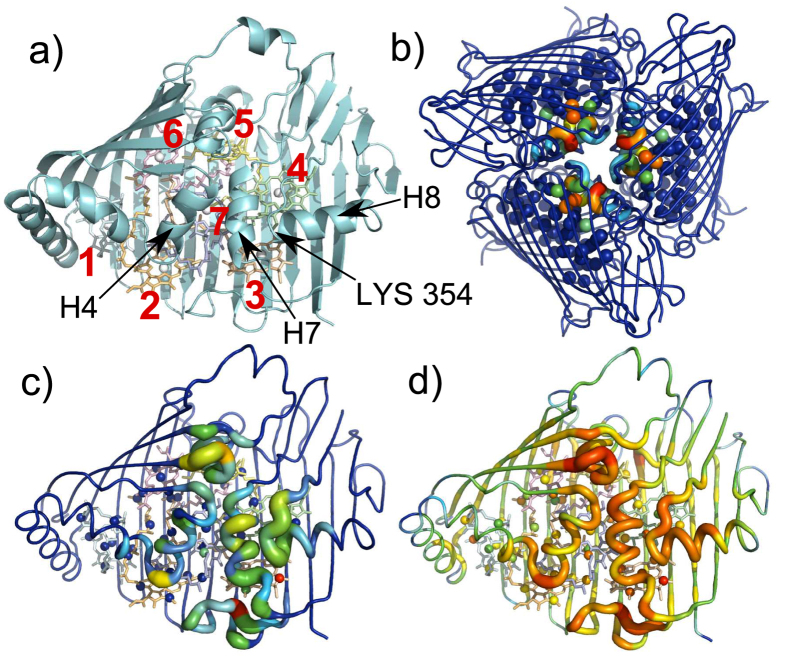
The structure of the FMO complex, coloured according to the spatial distribution of the high frequency NMs. (**a)** FMO monomer with the seven pigments, LYS 354 and helices 4, 7 and 8 labelled (H4, H7 and H8 respectively). (**b)** FMO trimer coloured according to participation in the highest frequency NM. (**c)** FMO monomer coloured according to average participation in the 20 highest frequency NMs. (**d)** FMO monomer coloured according to node degree. Note that for (**c**) monomer 1 is shown although monomers 2 and 3 are the same to within 0.025% of the total amplitude whilst for (**d**) all monomers are identical.

We begin by using the nonlinear network model of FMO outlined in the methods section to compute the high frequency NMs. Several of these NMs are delocalised across the trimer, for example the highest frequency NM is split equally across the three monomers, with almost identical contributions on individual residues in each monomer (to within 0.03% of the total amplitude). The amplitudes squared of the highest frequency NM on each node are plotted in Fig. 1b and the largest component on any single node is localised on residue LYS 354 (3.6% of the total amplitude is found on LYS 354 in each monomer). LYS 354 is highlighted in Fig. 1a. It lies on helix 8 and forms an inter-monomer salt bridge with ASP 306 and close hydrophobic contact with PHE 304, which is close to helix 7 on the neighbouring monomer. There are also large components on the pigments, the largest being the 7.4% localised on pigment 3 (split across the 8 pigment nodes). The amplitude of the NM squared on each node averaged over the 20 highest frequency NMs is shown in Fig. 1c. The node degree (i.e. the total number of connections each node makes) is plotted in Fig. 1d. We note that the total number of connections made by pigment atoms for pigments found in the core of the complex (pigments 3, 4, 5, 6 and 7) are higher than those found near the surface of the protein (pigments 1 and 2). Interestingly, in general the high frequency NMs tend to have strong amplitudes on nodes with high connectivities; Supplementary Fig. 1 plots the amplitude of the NM squared on a node averaged over the 20 highest frequency NMs as a function of the node’s degree. Since DBs emerge from the high frequency NMs, this is in keeping with the result of Piazza *et al.* that highly connected protein regions are always the areas in which DBs form^27^.

In order to study the protein’s dynamics we excite the highest frequency NM with a total energy of *E*_0_ = 40 kcal/mol and then simulate the molecular dynamics, as described in the methods section. This initial energy is typical when using the NNM to search for stiff nonlinear modes, and in line with values used by other authors^29^,^40^. The highest frequency NM is chosen following work in the literature which suggests that localised DB modes can emerge from a subset of high frequency NMs^26^,^28^. In nature, local perturbations to the local environment (caused by electronic photophysics or thermal fluctuations^30^ for example) could promote the random onset of these DB modes, which then harvest the relatively modest amount of 40 kcal/mol from the total energy of the system.

**Figure 2:**
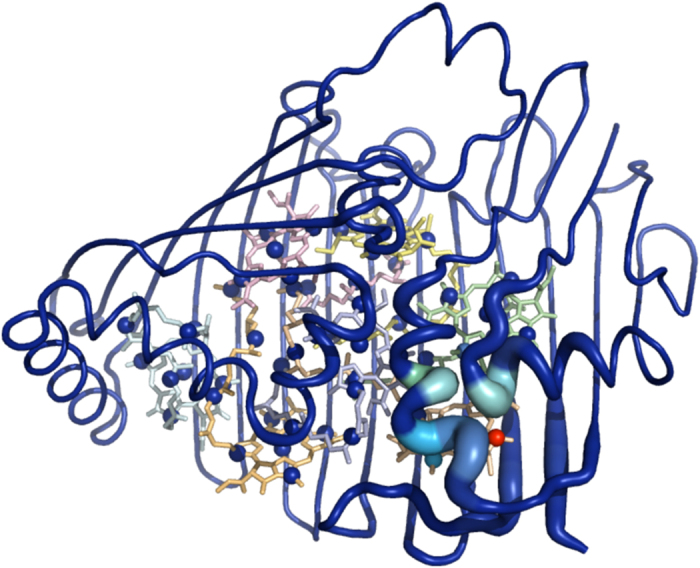
Monomer 3 coloured according to its average displacement during the MD simulation. The average displacement was calculated between 100 ps to 1 ns, following excitation of the highest frequency NM at t = 0.

We set *k*_2_ = 10 kcal/mol/Å^2^ and *k*_4_ = 10 kcal/mol/Å^4^, following Piazza *et al.*^26^,^30^, as described in the Methods section. Although the highest frequency NM is split equally across the three monomers, as described above, after 100 ps of the molecular dynamics simulation 72.8% of the energy is found on monomer 3. In Fig. 2 we plot monomer 3 of the FMO complex coloured according to the average displacement during the MD simulation from 100 ps to 1 ns. We observe considerable localisation, in particular pigment 3 exhibits the largest displacement and we also observe substantial vibrational motion of helices 7 and 8. Hence the nonlinear modes spontaneously break the symmetry of the high frequency delocalised NMs of the trimer. We note that which monomer the energy localises on is highly dependent on the initial excitation conditions, indicating competition between the monomers. Exciting a slightly lower frequency NM (the fourth highest frequency NM) leads to energy localisation at the interface of two monomers, as shown in the SI (the original normal mode is symmetric over all three monomers). Exciting a lower energy NM dissipates the energy approximately evenly across the structure, with no significant symmetry breaking.

**Figure 3:**
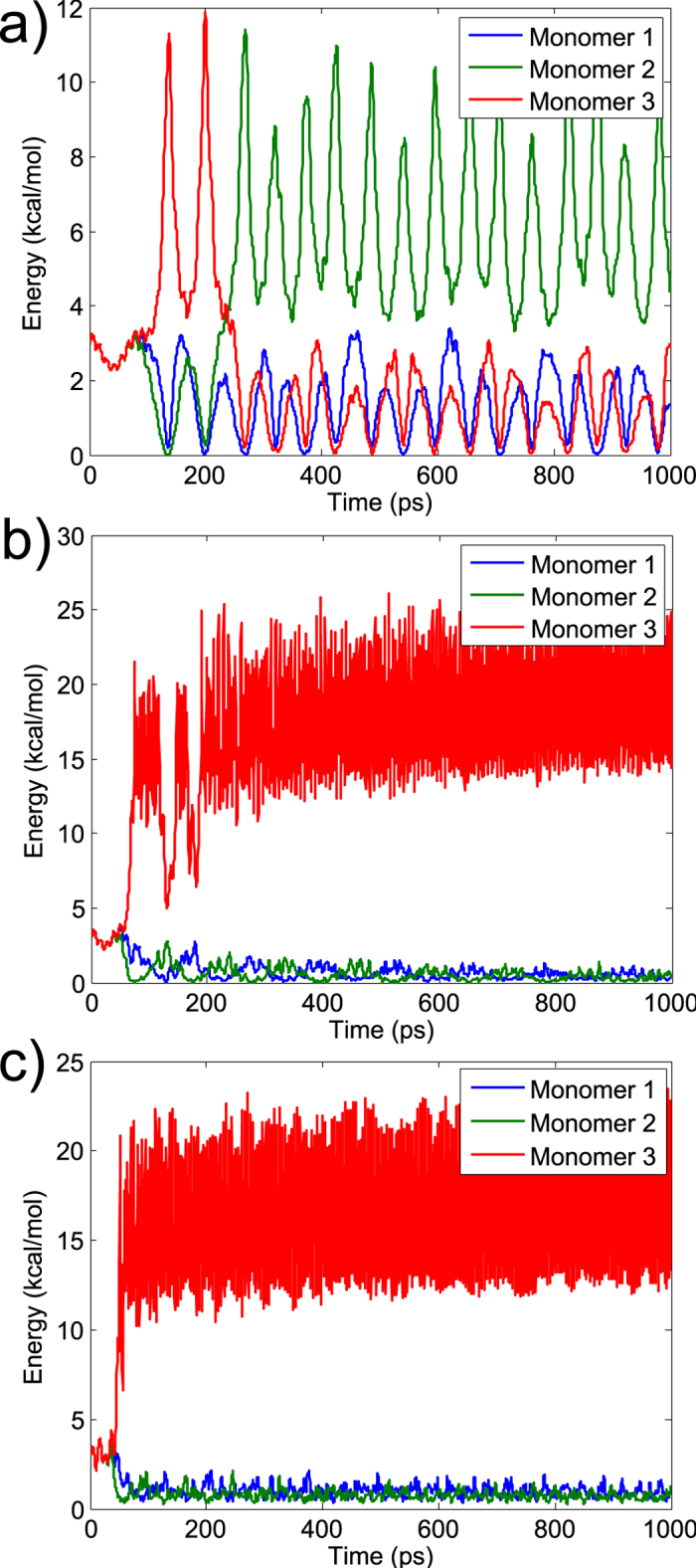
The total energy on pigment 3 following excitation of the highest frequency NM with 3 different values of anharmonicity. Results are shown over the first 1 ns. **a**, **b** and **c** plot results for *k*_4_ = 5, 10 and 15 kcal/mol^4^ respectively.

To study the localisation process in more detail, in Fig. 3 we plot the total energy on pigment 3 on each monomer over time, following excitation of the highest frequency NM, for three different values of anharmonicity: *k*_4_ = 5, 10 and 15 kcal/mol/Å^4^. As the anharmonicity increases, the localisation of energy on pigment 3 occurs more quickly, and vice versa. For *k*_4_ = 5 kcal/mol/Å^4^, we observe energy transfer between pigment 3 molecules on the three monomers (note that this continues at later times- for example after 1300 ps the energy is transferred to pigment 3 on monomer 1). Increasing/decreasing the excitation energy has a similar effect on the dynamics to increasing/decreasing the anharmonicity, for further details see Supplementary Fig. 2. Crucially, the localisation we observe around pigment 3 on a single monomer appears to be a fairly generic phenomenon, which occurs over a broad range of excitation energies and anharmonicity values. The energy remains localised for long times relative to excitation energy transfer in the FMO complex. We note that the energy is localised near to the core pigments, rather than at the surface of the protein. Since DBs arise as extensions of the highest frequency NMs by moving out of the phonon band^27^, this is in line with our observation that the high frequency NMs have substantial weight in these regions.

In other proteins, DBs have been found to localise in functional areas, such as hinges^30^, and have been speculated to play a crucial role in inducing conformational changes. From our results, the remarkable ability of the FMO complex to harvest energy from multiple spatial locations and localise it on and around the core pigments could have important consequences for light harvesting processes. Pigments 3 and 4 have been shown to act as the energy sink for FMO and funnel energy excitations to the reaction centre^41^,^42^, therefore vibrational energy transfer to this region is particularly interesting. Helices 7 and 8 have also been shown to be important in directing energy transport towards pigments 3 and 4 (the helix dipoles red shift the site energies)^42^. The functional significance of these intriguing results is unclear at present and deserves further investigation. We note that single localised modes have been shown to affect excitation energy transfer^12^, but we do not explore this further here.

We calculate the power spectrum of the x-displacement of the node in pigment molecule 3 with the highest energy (on the monomer with the highest energy), taken over a 300 ps period beginning after 300 ps of the molecular dynamics (not shown). For *k*_4_ = 10 kcal/mol/Å^4^, the main peak has a frequency of 104.8 cm^−1^, which is approximately 5.4 cm^−1^ higher than the frequency of the highest energy normal mode at 99.4 cm^−1^ (similar results were obtained for displacements in the y and z directions). From work by Juanico *et al.* and Piazza *et al.*^26^,^27^, this gap between the top of the harmonic spectrum and the frequency of the oscillations observed suggests the presence of a DB. The strong localisation of energy discussed above is also typical of DBs. For *k*_4_ = 5 kcal/mol/Å^4^ the main peak in the power spectrum shifts down to 100.1 cm^−1^. When *k*_4_ = 15 kcal/mol/Å^4^, we find the main peak is at the higher frequency of 106.7 cm^−1^ (in this case we take the trajectory between 100 ps and 400 ps).

In Fig. 3 we observe both high and low frequency oscillations in the energy on pigment 3 over time. By taking the Fourier transform of the total energy on pigment 3 between 300 and 1300 ps, we find that the main high frequency peaks lie at 200.3, 210.9 and 215.4 cm^−1^ for *k*_4_ = 5, 10 and 15 kcal/mol/Å^4^ respectively. In each case this is approximately twice the frequency of the main peak in the power spectrum of the trajectory. We also observe low frequency oscillations, corresponding to peaks in the Fourier transform of the total energy on pigment 3 at 0.6, 6.3 and 7.1 cm^−1^ for *k*_4_ = 5, 10 and 15 kcal/mol/Å^4^ respectively. These low frequency oscillations are a result of the nonlinear term in equation 1, however their exact origin remains unclear and requires further investigation. Picosecond timescales are known to be important for energy transfer in FMO, for example experimental observations by Zigmantas *et al.*^43^ suggested a 17 ps timescale for excitation energy transfer between the lowest energy FMO state and the reaction centre. Low frequency oscillations such as those observed prominently in Fig. 3a could therefore provide alternative energy transfer pathways, perhaps enabling ‘trapped’ energy to be redistributed.

Similar results are obtained when other high frequency NMs are excited, namely the energy becomes localised on a specific pigment or residue with high connectivity and a DB forms with a frequency which lies above the top of the harmonic spectrum. For example, when the fourth highest frequency NM is excited the energy becomes localised on residue GLY 231, which is found in the core of the complex near to helix 4 (the second and third highest frequency NMs are similar to the highest frequency NM, due to symmetry). Note that in this case we excite the complex with *E*_0_ = 50 kcal/mol since *E*_0_ = 40 kcal/mol does not lead to localisation on any single node within the first 1 ns. This is in contrast to simulations where low energy NMs are excited and the energy is dissipated across the protein over time. Examples of these dynamics are shown in the SI.

Having explored the intriguing possibility of nonlinear DB modes in FMO and analysed their properties and origins, we now consider how the spatial structure of these modes might affect FMO’s optoelectronic properties. Several models of FMO’s optical spectra, including non-linear spectra, have been presented previously in the literature^6^,^25^,^36^,^41^,^44^, but to our knowledge none have included non-trivial spatial distributions of modes based on NNM results. Various studies indicate that correlated dynamical fluctuations (as revealed by NMA) seem to have a limited impact on excitonic transfer dynamics in light harvesting complexes when all other effects of the bath are accounted for^25^,^36^,^45^,^46^, but their presence might still affect the optical spectra because of the ways in which the spatial properties of the coupling affect the vibronic Hamiltonian.

Here we consider the linear absorption, linear dichroism and circular dichroism spectra, which are frequently used to examine molecular systems. As discussed in the methods section, we use the *ab initio* pigment energies and couplings obtained by Cole *et al.*^33^ to calculate the spectra, however crucially we also include a single discrete mode in our calculations in order to approximate the effect of a DB. This mode is treated at the same level as the electronic states. Above we showed that exciting the highest frequency NM leads to large displacements of pigment 3 as well as helices 7 and 8. We are not aware of any studies explicitly studying the variation in pigment site energies with helix displacements, but the proximity of helices 7 and 8 to pigments 4 and 7 suggests that their site energies are also likely to be modulated by the DB. Other pigments are not expected to be affected. Therefore we couple the mode to pigments 3, 4 and 7 only. In practice the strength of the coupling to different pigments may vary but as a first approximation we set the Huang Rhys factor S = 0.3 for all three pigments. We note that some previous models of spectral functions do include discrete modes of 180 cm^−1^ coupling independently and identically to all pigments^41^. Therefore for comparison we also calculate the spectra for a single discrete mode coupling to all seven pigments in our ab initio model.

The frequency of the DB is unknown (it is determined by *k*_2_ and *k*_4_ in our model), but from the literature high frequency protein normal modes (excluding intramolecular pigment modes) might be expected to lie between approximately 100 and 200 cm^−1 ^19,26,27. Modes have been observed experimentally with *ω* ≈ 70 cm^−1^, *ω* ≈ 117 cm^−1^ and *ω* ≈ 180 cm^−1 ^47. Given this uncertainty in the exact mode frequency, in our calculations we consider two mode frequencies as examples: *ω* = 100 and 180 cm^−1^. We note that the mode with *ω* ≈ 180 cm^−1^ is generally considered to be a pigment mode^12^,^47^, however as far as we are aware the intriguing possibility that this mode and the modes close to 100 cm^−1^ could be at least in part due to protein vibrations has not been ruled out. Interestingly, work by Leitner *et al.* suggested that protein modes might enhance chromophore vibrations in photoactive yellow protein^48^. Note that we require *k*_2_ = 30 kcal/mol^2^ and *k*_4_ = 30 kcal/mol^4^ for the top of the harmonic spectrum to lie around 180 cm^−1^ and previous work showing the high level of stiffness in FMO suggests that this is not unreasonable^32^. The dynamics following excitation of the highest frequency NM with *k*_2_ = 30 kcal/mol^2^ and *k*_4_ = 30 kcal/mol^4^ are qualitatively similar to those obtained previously, leading to energy transfer to pigment 3, as shown in the SI (in this case we used *E*_0_ = 120 kcal/mol to obtain localisation).

**Figure 4:**
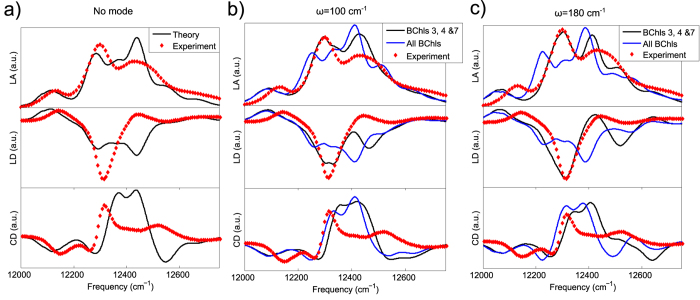
Simulated and experimental optical spectra. LA, LD and CD spectra are shown for (**a**) calculations with no mode (black line) and (**b**) for a mode with *ω* = 100 cm^−1^ coupled firstly to pigments 3, 4 and 7 only (black line) and secondly to all pigments equally (blue line). (**c**) As for (**b**) but with *ω* = 180 cm^−1^. In all parts, experimental spectra are shown in red for comparison^49^.

Figure 4 plots optical spectra for *ω* = 100 cm^−1^ and *ω* = 180 cm^−1^ coupled to pigments 3, 4 and 7, as suggested by the NNM dynamics. We also plot the optical spectra with no mode (as calculated by Cole *et al.*^33^) and spectra with modes coupled to all pigments (not just pigments 3, 4 and 7), for comparison. All calculated spectra are compared to experimental results for *P. aestuarii* obtained at 77 K^49^ (plotted in red). Note that the intensities of the calculated spectra were scaled to match the experimental results.

For both *ω* = 100 cm^−1^ and *ω* = 180 cm^−1^, coupling the discrete mode to all seven pigments (as described in previous studies^41^) does not improve the calculated spectra’s agreement with the experimental results. However, when we use the pattern of displacements corresponding to the nonlinear modes and couple the mode to pigments 3, 4 and 7 only, for both *ω* = 100 cm^−1^ and *ω* = 180 cm^−1^, we do observe some improvement in both the linear absorption and linear dichroism spectra (in comparison to experimental results). In particular, the peak around 12300 cm^−1^ in the linear absorption, which was underestimated in calculations with no mode becomes more prominent. Meanwhile the peak around 12450 cm^−1^, which was overestimated in the calculations with no mode, is reduced. In the linear dichroism spectrum, the peak at 12300 cm^−1^ was underestimated in the original calculations. When the mode is included this peak is much deeper. The linear dichroism spectra around 12400 cm^−1^ are also much closer to experimental results with the mode, particularly when *ω* = 180 cm^−1^. We note that whilst in equation 2 we have assumed linear coupling to the mode, when an anharmonic energy shift was included we did not notice any substantial spectral changes. Therefore we conclude that nonlinearity in the vibrational Hamiltonian gives negligible effects, and the key factor in determining the spectra is the spatial pattern of the vibrational modes.

Note that the coupling of the mode to pigment 3 lowers the energy of the lowest energy exciton, which red shifts the low energy parts of the spectra, slightly worsening the agreement with the experimental results in that region. Meanwhile, the agreement of the simulated CD spectra with experimental values is not as good as the results for the linear spectra and shows no sign of improvement when a mode is included, regardless of which pigments the mode couples to. These remaining discrepancies between theory and experiment could be caused by errors in the *ab initio* pigment site energies. The relative site energy of pigment 3 is lower than the result obtained by Adolphs *et al.* for example^41^. Further work is required to overcome assumptions made in the calculation of the *ab initio* site energies, which are discussed in detail elsewhere^33^. Nevertheless, with these improvements we would expect the methods described here to be applicable to the interpretation of 2D electronic spectra of the FMO complex, as well as to the study of other pigment-protein complexes.

## Conclusions

To our knowledge, this work is the first to apply the nonlinear network model to study the archetypal light-harvesting complex, FMO. This computationally inexpensive approach allows us to investigate FMO’s collective protein modes, which have been implicated in the energy transfer processes, at a microscopic level. Our model also incorporates an anharmonic term which enables us take a first step towards capturing the nonlinear effects that previous work shows can play a role in protein dynamics^22^,^23^,^50^.

We find that the high frequency NMs can be delocalised across the trimer and exhibit strong components on the core pigments and on certain residues with high connectivity. When high frequency NMs are excited, we observe energy localisation in biologically relevant areas of the protein. For example, exciting the highest frequency NM leads to energy localisation around pigment 3 and helices 7 and 8, which are expected to modulate the site energies of pigments 4 and 7. By studying the power spectrum we show that a DB mode has been formed and similar results can be obtained following excitation of other high frequency NMs. These non-linear DB modes offer the potential for intriguing alternative energy transfer routes. In particular they could play a role in localising and storing excess energy around pigments 3 and 4, which are known to funnel energy excitations towards the reaction centre. Future work should focus on the details of these processes, both experimentally and by establishing more accurate theoretical models. The ability of DBs to transfer energy around the protein could also lead to a non-equilibrium environment which is temporally spatially dependent; in other words the environment has a spatial dependence which changes over time. These dynamics might offer new ways to co-ordinate excitation energy transfer. This type of environment has received little attention to date and could open up exciting possibilities for device design.

Having established the existence of potentially biologically important DBs in our system, we investigated the effect which these localised modes might have on the optical spectra which were calculated by Cole *et al.*^33^ using *ab initio* site energies and couplings. To do this we approximated the DB as a single mode, localised on pigments 3, 4 and 7 with example frequencies of *ω* = 100 or 180 cm^−1^. We incorporated this mode into optical spectra calculations on an equal footing with the electronic energy levels. Substantial improvements in the LA and LD spectra were obtained, particularly around the 12300–12450 cm^−1^ region. We note that this is in contrast to the effect of incorporating very low frequency modes, which previous work found did not affect the spectra^32^. Nonetheless there is room for improvement, in particular the lowest energy exciton is lower than observed experimentally and the circular dichroism spectra still show large differences from the experimental results. Further work is needed to investigate the cause of these discrepancies.

## Additional Information

**How to cite this article**: Morgan, S. E. *et al.* Nonlinear network model analysis of vibrational energy transfer and localisation in the Fenna-Matthews-Olson complex. *Sci. Rep.* **6**, 36703; doi: 10.1038/srep36703 (2016).

**Publisher’s note:** Springer Nature remains neutral with regard to jurisdictional claims in published maps and institutional affiliations.

## Supplementary Material

Supplementary Information
