# Supplementary material for: Nonlinear network model analysis of vibrational energy transfer and localisation in the Fenna-Matthews-Olson complex
Source: Sci Rep. 2016 Nov 9;6:36703. doi: 10.1038/srep36703 (PMC5101523; doi:10.1038/srep36703)
Supplement: Supplementary Information [file srep36703-s1.pdf]

Supplementary Information for: Nonlinear network model analysis of vibrational energy transfer and localisation in the Fenna-Matthews-Olson complex

Sarah E. Morgan*[1], Daniel J. Cole[1,2], and Alex W. Chin[1]

[1]Theory of Condensed Matter group, Cavendish Laboratory, University of Cambridge, JJ Thomson Avenue, Cambridge CB3 0HE, United Kingdom

[2]School of Chemistry, Newcastle University, Newcastle upon Tyne, NE1 7RU, United Kingdom

Contents

1 Relationship between a node's participation in the 20 highest frequency NMs and its degree 2
2 Dynamics with different intial excitation energies 3
3 Dynamics following excitation of alternative NMs 4
4 Dynamics with $k_2 = 30$ kcal/mol/$\mathring{A}^2$ 6

1 Relationship between a node's participation in the 20 highest frequency NMs and its degree

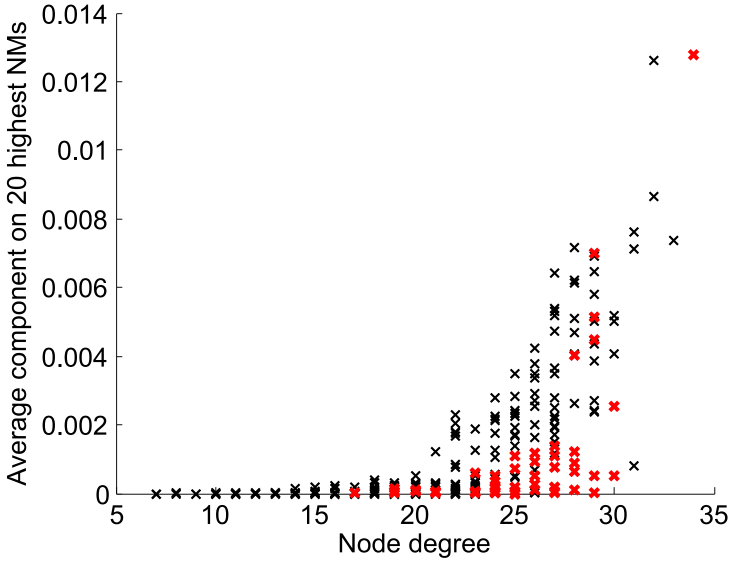

Supplementary Figure 1: The amplitude of the NM squared on a node averaged over the 20 highest frequency NMs as a function of the node's degree. The pigment nodes are higlighted in red.

2 Dynamics with different intial excitation energies

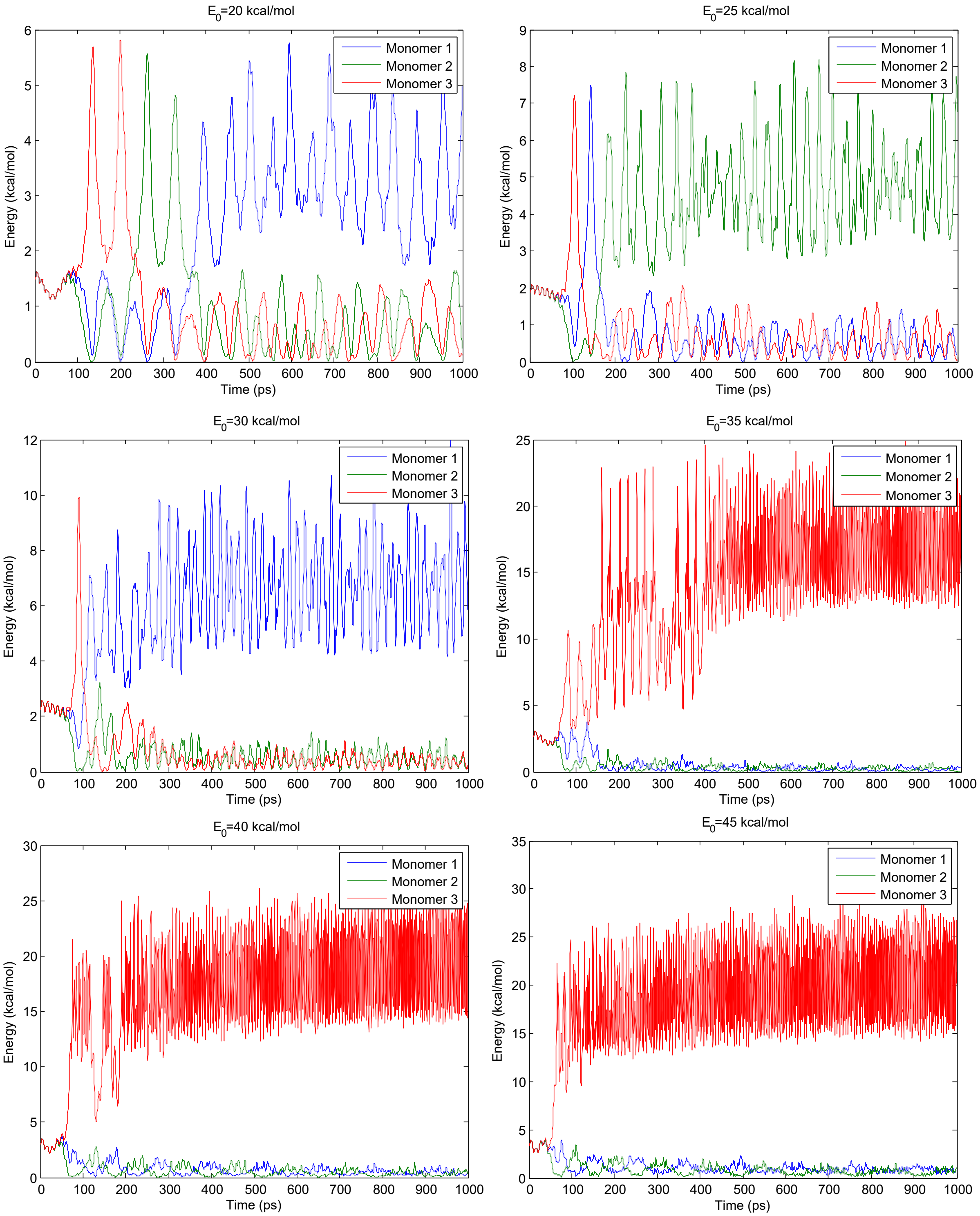

Supplementary Figure 2: The total energy on pigment 3 on each monomer over 1 ns following excitation of the highest frequency NM (NM 3726). Results are plotted with $k_2 = 10$ kcal/mol/Å^2, $k_4 = 10$ kcal/mol/Å^4 and initial energies of E_0 = 20, 25, 30, 35, 40 and 45 kcal/mol.

3 Dynamics following excitation of alternative NMs

As an example of exciting a different high frequency NM, we consider excitation of the fourth highest frequency NM- NM 3723 (the second and third highest frequency NMs are not delocalised across the whole trimer but have components on similar nodes within the monomer to the highest frequency NM, due to symmetry). Note that here we refer to the highest frequency NM as NM 3726 and the lowest frequency NM as NM 1. The FMO complex coloured according to the amplitude of NM 3723 squared is plotted in Supplementary Fig. 3. Note that in this case we excite the NM with $E_0 = 50$ kcal/mol to obtain localisation of the energy (exciting NM 3723 with $E_0 = 40$ kcal/mol does not lead to substantial energy localisaton within the first 2 ns). In Supplementary Fig. 4 we plot the energy on residue GLY 231 on monomer 3 and we observe that energy becomes localised on this residue after around 1100 ps. In Supplementary Fig. 5 b we plot the FMO complex coloured according to the average displacement during the MD simulation from 1100 ps to 2000 ps following excitation of NM 3723 (alongside the equivalent plot for the MD simulation following excitation of NM 3726 as described in the main text in part a). We find that the energy is localised at the interface between two monomers.

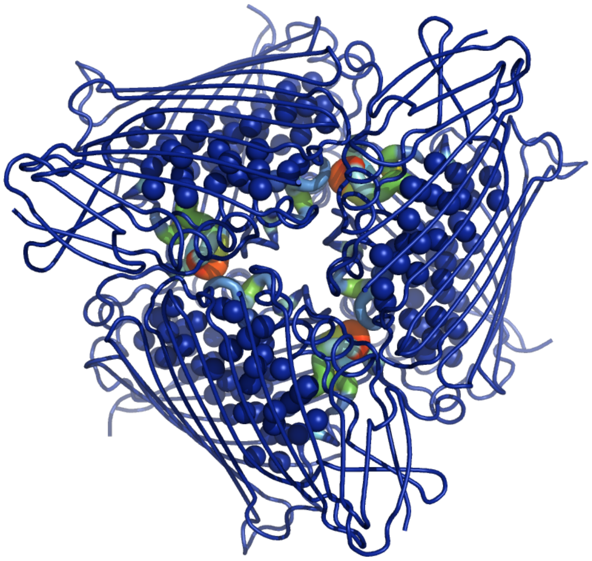

Supplementary Figure 3: The FMO complex coloured according to the amplitude of NM 3723 squared.

When lower frequency NMs are excited, the energy tends to become dissipated across the complex over time. For example after exciting NM 1206 (which has a frequency of 50.01 cm^{-1}) with $E_0 = 40$ kcal/mol, no individual particle has more than 0.7% of the total energy after 1 ns. The FMO complex coloured according to the average displacement during the MD simulation from 100 ps to 1 ns is plotted in Supplementary Fig. 5 c.

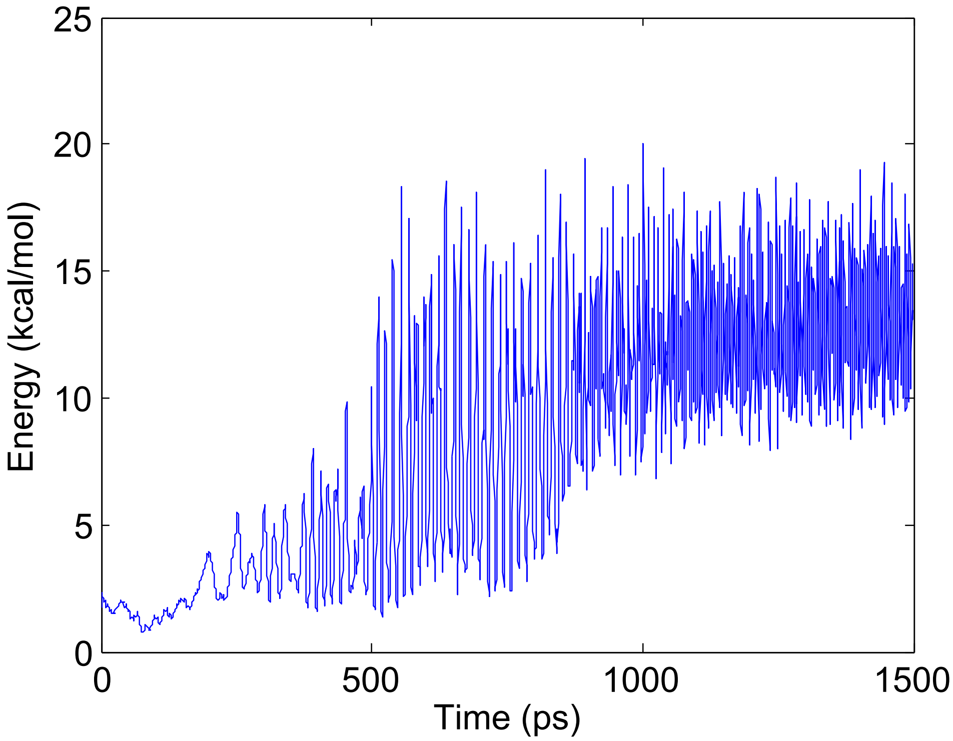

Supplementary Figure 4: The total energy on GLY 231 of monomer 3 over 1500 ps following excitation of the fourth highest frequency NM (NM 3723).

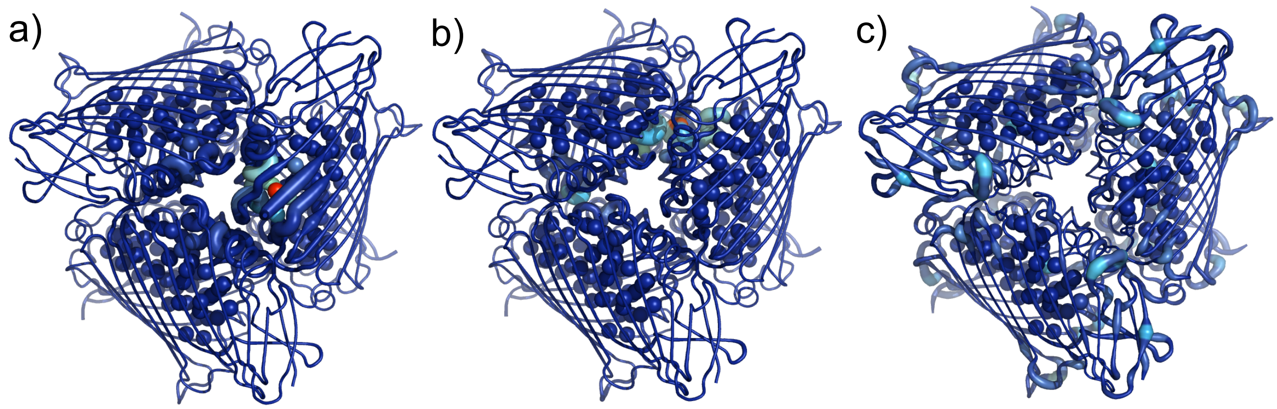

Supplementary Figure 5: The FMO complex coloured according to the average displacement during MD simulations following the excitation of NMs 3726, 3723 and 1206. The residues are coloured according to **a)** the average displacement during the MD simulation from 100 ps to 1 ns, following excitation of NM 3726 with $E_0 = 40$ kcal/mol, **b)** the average displacement during the MD simulation from 1.1 ns to 2 ns, following excitation of NM 3723 with $E_0 = 50$ kcal/mol, **c)** the average displacement during the MD simulation from 100 ps to 1 ns, following excitation of NM 1206 with $E_0 = 40$ kcal/mol.

4 Dynamics with $k_2 = 30$ kcal/mol/Å^2

In order for the frequency of a DB to lie around 180 cm^{-1}, we require a higher k_2 value than $k_2 = 10$ kcal/mol/Å^2 as used in the main text. For example, when $k_2 = 30$ kcal/mol/Å^2, the top of the harmonic spectrum lies at 172 cm^{-1} (from discussions in the main text, the DB frequency is expected to be approximately $5 - 10$ cm^{-1} higher). Dynamics for the case where $k_2 = 30$ kcal/mol/Å^2 and $k_4 = 30$ kcal/mol/Å^4 are shown in Supplementary Fig. 6. Here an initial energy of $E_0 = 120$ kcal/mol leads to the formation of a DB after approximately 100 ps. As before, the energy becomes localised around pigments 3, 4 and 7. The FMO complex coloured according to the average displacement during the MD simulation from 100 ps to 1 ns is shown in Supplementary Fig. 7 and is almost identical to the results with $k_2 = 10$ kcal/mol/Å^2 and $k_4 = 10$ kcal/mol/Å^4 in Supplementary Fig. 5 a.

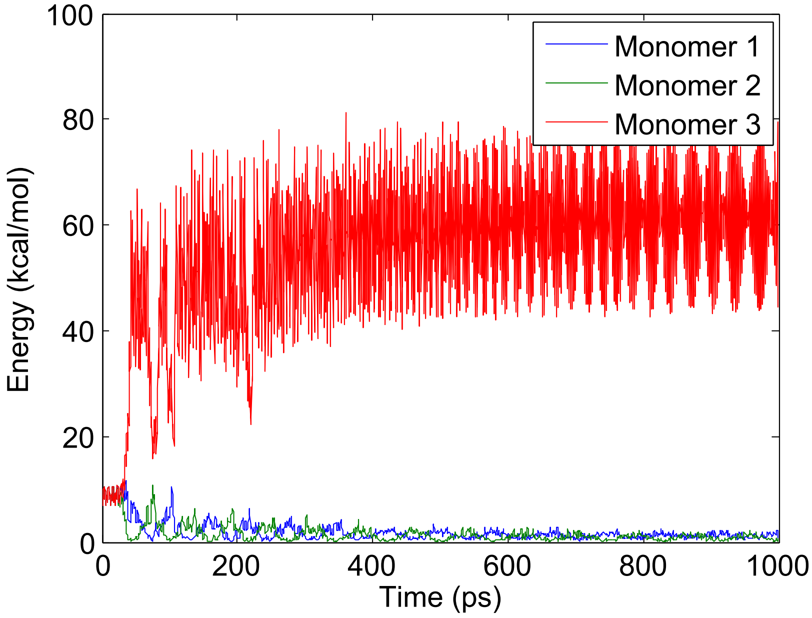

Supplementary Figure 6: The total energy on pigment 3 on each monomer over 1 ns following excitation of the fourth frequency NM (NM 3723). Results are plotted with $k_2 = 30$ kcal/mol/Å^2, $k_4 = 30$ kcal/mol/Å^4 and $E_0 = 120$ kcal/mol.

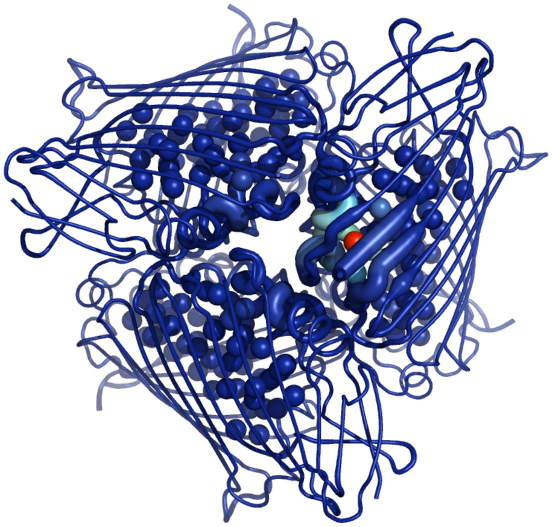

Supplementary Figure 7: The FMO complex coloured according to the average displacement during the MD simulation from 100 ps to 1 ns, following excitation of NM 3726. Results are plotted with $k_2 = 30$ kcal/mol/Å^2, $k_4 = 30$ kcal/mol/Å^4 and $E_0 = 120$ kcal/mol.
